# Effect of a suitable treatment period on the genetic transformation efficiency of the plant leaf disc method

**DOI:** 10.1186/s13007-023-00994-3

**Published:** 2023-02-15

**Authors:** Yufei Xia, Yuan Cao, Yongyu Ren, Aoyu Ling, Kang Du, Yun Li, Jun Yang, Xiangyang Kang

**Affiliations:** 1 National Engineering Research Center of Tree Breeding and Ecological Restoration, Key Laboratory of Genetics and Breeding in Forest Trees and Ornamental Plants of Ministry of Education, College of Biological Sciences and Biotechnology, Beijing Forestry University, Beijing, 100083 China; 2 State Key Laboratory of Tree Genetics and Breeding, Chinese Academy of Forestry, Beijing, 100091 China

**Keywords:** Differentiation culture, Leaf disk method, Transformation efficiency, Flow cytometry, EdU staining, Cell cycle

## Abstract

**Background:**

*Agrobacterium tumefaciens-*mediated leaf disc genetic transformation is an important way to achieve transgenics or gene editing. Ensuring stable and efficient genetic transformation is still an important problem in modern biology. It is assumed that the difference in the development status of genetic transformation cells of receptor materials is the main reason for the difference and instability of genetic transformation efficiency; the stable and efficient genetic transformation rate can be obtained by defining the appropriate treatment period of the receptor material and applying genetic transformation in a timely manner.

**Results:**

Based on these assumptions, we studied and established an efficient and stable *Agrobacterium*-mediated plant transformation system with hybrid poplar (*Populus alba* × *Populus glandulosa*, 84 K) leaves, stem segments and tobacco leaves as the research objects. There were differences in the development process of leaf bud primordial cells from different explants, and the genetic transformation efficiency was significantly related to the cell development stage of the in vitro cultured materials. Among them, the genetic transformation rate of poplar and tobacco leaves was the highest on the 3rd and 2nd day of culture, reaching 86.6% and 57.3%, respectively. The genetic transformation rate of poplar stem segments was the highest on the 4th day of culture, reaching 77.8%. The best treatment period was from the development of leaf bud primordial cells to the S phase of the cell cycle. The number of cells detected using flow cytometry and 5-ethynyl-2ʹ-deoxyuridine (EdU) staining, the expression of cell cycle-related protein CDKB1; 2, CDKD1; 1, CYCA3; 4, CYCD1; 1, CYCD3; 2, CYCD6; 1, and CYCH; 1 of explants, and morphological changes of explants can be used as indicators to determine the appropriate treatment period for genetic transformation.

**Conclusions:**

Our study provides a new and universal set of methods and characteristics to identify the S phase of the cell cycle and apply genetic transformation treatments at the appropriate time. Our results are of great significance for improving the efficiency and stability of plant leaf disc genetic transformation.

**Supplementary Information:**

The online version contains supplementary material available at 10.1186/s13007-023-00994-3.

## Background

Plant genetic transformation is a key step in molecular design breeding, including genetic engineering and genome editing [1]. It can not only be used to identify gene functions but also to improve short plate traits of plant varieties, especially for genetic improvement of target traits of tree varieties with a long breeding generation cycle, which can significantly shorten the breeding cycle [2]. Genetic transformation mainly depends on the plant regeneration ability and the efficiency of gene introduction or editing of plant cells [3]. *Agrobacterium tumefaciens*-mediated leaf disc transformation is a plant genetic transformation method developed by Horsch et al. [4, 5]. The improved leaf disc method includes stem segments, petioles, hypocotyls, cotyledons, and other explant materials [6, 7]. This method introduces foreign DNA fragments or gene editors carried by Ti plasmids, Ri plasmids, and other carriers into host cells through *Agrobacterium* infection to study related gene functions or realize plant breeding. It is the most widely used genetic transformation technology and method, promoting the research progress of plant molecular breeding [1, 8, 9].

Currently, many plants have achieved genetic transformation, most of which have been successfully transformed by *A. tumefaciens*-mediated leaf discs [10–12]. Despite this, the low transformation efficiency of the plant leaf disc method is still a bottleneck problem that restricts transgenics and gene editing [13–16]. Among these, tobacco, as a model plant of the plant transgenic receptor, was transformed by different *Agrobacterium* strains, and the transformation efficiency was between 8.3 and 20% [17]. By transforming different poplar explants, the transformation efficiency was 21–36.3% [18]. By optimizing the concentration and infection time of *A. tumefaciens*, the highest transformation efficiency of the poplar leaf disc method was about 25%, and the lowest was only about 5% [19]. It was also reported that the transformation efficiency of poplar leaves infected with *A. tumefaciens* was only 32.18% after 2 days of culture [20]. Tomato was transformed by the leaf disc method with *A. tumefaciens* solution, and the transformation efficiency was 0.79–2.28% [21]. The transformation efficiency of different tomato varieties ranged from 0 to 11.13% [22]. Another solanaceous plant, potato, had a transformation efficiency of 6–17% with leaf discs as explants, and different potato varieties had different transformation efficiencies [23]. Soybean was the natural host of *A. tumefaciens*, but the genetic transformation of different varieties was quite different, and the transformation rate was generally 0.2–10.0% [24–27]. From more than 100 soybean genotypes, researchers found that ‘Peking’ was more sensitive to *A. tumefacience* [28]. Few varieties have obtained a transformation rate higher than 10%, such as the ‘YC-1’ and ‘YC-2’ varieties, whose transformation rate reached about 14.7% [29]. Maize, wheat, and other monocotyledons initially used immature embryos as genetic transformation receptor materials, and the transformation efficiency was greatly affected by genotype, ranging from 5.5 to 30.6% and 1.7 to 10.5% [30]. Although the transformation rate can be improved through genotype screening, *Agrobacterium* transformation, and optimization of culture conditions, the problem of unstable plant transformation efficiency has not been fundamentally solved [30, 31].

Many studies have shown that different plant species, different genotypes within the same species, different explants of the same species, and different development stages of explants have a great impact on transformation efficiency, and the rate of genetic transformation can be improved when explants are cultured to a suitable state of cell development [6, 7, 19, 31–34]. When the genetic transformation of barley was carried out, the transformation rate of barley cultured on differentiation medium for 1 day was higher than that of the uncultured, and the transformation rate dropped sharply after 1 day [35]. Although the research results for some species were different, and the conclusions were also inconsistent [32, 36], there was a correlation between the explant development status and the transformation rate of explants [37]. The optimal culture time of explants was 1–4 days [35, 38–40]. Competent cells in the division phase or active metabolism were easily infected by *A. tumefaciens* and then transformed. Culture on differentiation medium could promote the division of receptor cells and improved the metabolic activity of cells, and it was easy to integrate foreign DNA to improve the transformation rate [41, 42]. However, it may be too general to explain only from promoting the division of receptor cells and improving the metabolic activity of cells. It is not clear whether cultivating receptor materials on a differentiation medium before genetic transformation is the fundamental reason for improving the transformation rate, nor does it propose a set of identification techniques suitable for different transformation materials in terms of relevant reasons.

For different species, different genotypes of the same species, different explants of the same genotype, and receptor materials of the same explant at different developmental stages, there must be some differences in the development of transformed cells when transformation is applied, which may be the main reason for the differences and instability of different genetic transformation research results. In the process of genetic transformation, an efficient and stable genetic transformation rate would benefit from making the transformed cells reach the appropriate treatment period through differentiation culture. Based on these assumptions, an efficient and stable *Agrobacterium*-mediated plant transformation system was established using 84 K poplar and tobacco as materials. We observed the morphology and cytology of poplar leaves, stem segments and tobacco leaf explants cultured on differentiation medium at different times, studied the cell development process through flow cytometry, EdU staining, and key gene expression, and finally conducted genetic transformation. The purpose was to analyze the relationship between the cell development status of receptor materials of different tissue types and genetic transformation efficiency, determine the appropriate treatment period, clarify the mechanism of receptor materials and cell development status affecting plant genetic transformation efficiency, and propose a matching instant discrimination method to provide technical support for efficient and accurate genetic transformation and gene editing of plants.

## Materials and methods

### Plant materials

Hybrid poplar 84 K and tobacco were used to obtain explants for the transformation study. Stems of 84 K were propagated from microcuttings in bottles and cultured on rooting medium (RM) containing 1/2 Murashige and Skoog (MS), 30 g L^−1^ sucrose, 6 g L^−1^ agar, 0.02 mg L^−1^ naphthylacetic acid (NAA), and 0.05 mg L^−1^ indolebutyric acid (IBA), and stems of tobacco were cultured on rooting medium (RM) containing MS, 30 g L^−1^ sucrose, 6 g L^−1^ agar and 0.4 mg L^−1^ indolebutyric acid (IBA). They were grown in an artificial climate chamber (25 °C, 16 h/8 h light/dark photoperiod, and 55% relative humidity).

### Differentiation culture of acceptor material

The 3rd–5th leaves of 1-month-old 84 K poplar and tobacco tissue culture seedlings were cut perpendicular to the veins with a blade, and the 3rd–5th internodes of the 84 K poplar were cut off. They were placed in poplar differentiation medium (MS, 30 g L^−1^ sucrose, 6 g L^−1^ agar, 0.05 mg L^−1^ NAA, and 0.5 mg L^−1^ 6-Benzylaminopurine (6-BA)) and tobacco differentiation medium (MS, 30 g L^−1^ sucrose, 6 g L^−1^ agar, 0.01 mg L^−1^ NAA, and 0.2 mg L^−1^ 6-BA) and cultured for 1, 2, 3, 4, and 5 days. The culture conditions were as follows: the culture temperature was 25 ± 2 °C, the light was 2000 lx, and the light cycle was 16 h light/8 h dark.

### Observation of phenotypic characteristics of in vitro materials with different differentiation culture time

After removing the agar from the poplar leaves or stems and tobacco leaves cultured in vitro, they were placed under a stereo microscope (Olympus SZX12) for observation. Photographs were taken, and the callus development status at the incision site of the in vitro culture material treated with five different differentiation culture times (1, 2, 3, 4, and 5 days) and control treatments (differentiation culture 0 days) was recorded.

### Cytological observation of receptor material after differentiation culture

The acceptor material was cultured on differentiation medium for 0, 1, 2, 3, 4, and 5 days, and then the incision was cut with a razor blade and fixed with the fixative FAA for 24 h. The tissues were dehydrated, dipped in wax, and embedded, and the paraffin block was placed in a paraffin microtome (RM2016, Leica) for sectioning to a thickness of 4 μm. After staining with safranine O-fast green, the samples were observed and photographed under a microscope (Nikon ECLIPSE CI, Japan).

### Flow cytometry

We detected cell cycle using flow cytometry (BD FACSCalibur, USA) [43, 44]. According to the previous method [45], approximately 0.5 g of minced young leaves or stem segments were placed in 1 ml of nuclear extraction buffer (45 mM MgCl_2_ 6H_2_O, 30 mM sodium citrate, 20 mM MOPS, 1% (v/v) Triton X-100, pH 7.0) in a 55-mm petri dish and filtered through a 30-μm nylon mesh. Nuclei were stained with 80 μL DAPI (5 mg/mL) for 10 min, and three samples were collected for each treatment [46, 47]. The peak maps were analyzed using Cyflow^®^ Ploidy Analyzer (Partec PAS, Germany), and the proportion of G1, S, and G2/M phases in the peak maps was identified using modfitLT software [43, 45].

### 5-Ethynyl-2’-deoxyuridine (EdU) staining

The 84 K poplar leaves, stem segments and tobacco leaves were cultured on differentiation medium for 1, 2, 3, 4, 5, and 6 days, and then placed on differentiation medium containing 10 μM EdU for 24 h. After material incubation was completed, the material was washed three times with Phosphate Buffered Saline (PBS). It was then fixed with 4% paraformaldehyde, treated in the dark for 30 min, washed three times with PBS, and treated with 0.5% Triton X-100 for 15 min to promote penetration. The samples were then processed according to the instructions of YF®488 Click-iT EdU Imaging Kits (BN16015, Biorigin, Beijing), and finally photographed and analyzed under a laser confocal microscope (Leica TCS SP8; Leica, Wetzlar, Germany). An argon ion wavelength of 488 nm was employed for EdU and chlorophyll. Fluorescence was detected at 495–515 nm for EdU and at 650 nm for chlorophyll [48].

### RNA-seq experiment and data analysis

Thirty 84 K poplar plants with similar growth status were selected, the leaves located at the 3rd–5th leaf position were cut off, and three to four wounds were cut perpendicular to the leaf veins with a blade and placed on differentiation medium for culture. After 0, 1, 2, 3, 4, and 5 days of differentiation culture, the leaves were harvested, put into cryopreservation tubes, and then snap frozen in liquid nitrogen. RNA extraction, library construction, and sequencing were performed at Nuohezhiyuan Technology Co., Ltd. (Beijing, China). Clean reads were precisely aligned to the 84 K poplar genome using HISAT2 software [49]. FeatureCounts v1.5.0-p3 was used to count the reads mapped to each gene. The FPKM of each gene was then calculated based on the length of the gene and the read count mapped to this gene. The data of 1, 2, 3, 4, and 5 days of differentiation culture were compared with the control (0 days of culture), and differentially expressed genes (DEGs) were identified. Differential expression analysis of the two groups was performed using the DESeq2 R package (1.20.0). Genes with an adjusted *P* ≤ 0.05 found by DESeq2 were assigned as differentially expressed.

### Gene ontology (GO) enrichment analysis

GO enrichment analysis of DEGs was implemented using the clusterProfiler R package. GO terms with corrected *P* < 0.05 were considered significantly enriched as DEGs.

### RNA extraction and quantitative real-time polymerase chain reaction (RT-qPCR) analysis

Total RNA was extracted from plant tissues using a Plant Total RNA Extraction Kit (Tiangen, China, Cat DP432). cDNA was synthesized using a cDNA synthesis kit (Tiangen, China, Cat. KR106) following the protocols. RT-qPCR assays were performed using TransStart Top Green qPCR SuperMix (TRANSGEN, China, Cat. AQ132-22) [50]. Three biological replicates were performed for each tissue. Gene-specific primers from qPrimerDB (https://biodb.swu.edu.cn/qprimerdb) [51]. The 2^−ΔΔCt^ method was used to calculate relative gene expression levels [52]. The primers are shown in Additional file 6: Table S1.

### *Agrobacterium*-mediated genetic transformation and genetic transformation efficiency statistics

Genetic transformation of the in vitro materials was conducted using the *Agrobacterium* GV3101 strain carrying the expression vector pBI121 containing the GFP reporter gene. A single colony of *Agrobacterium* carrying the pBI121 binary vector was inoculated into 50 ml of YEB liquid medium (0.25 g sodium chloride, 0.5 g yeast extract, and 0.5 g peptone) supplemented with 50 mg L^−1^ kanamycin (Kan) and 25 mg L^−1^rifampicin (Rif). Liquid suspension culture was carried out until the bacterial liquid was uniform and the OD_600_ of the bacterial liquid reached 0.6–0.8; that is, the infecting bacterial liquid was obtained.

The cultured poplar leaves, stem segments and tobacco leaves were placed in the infection bacteria solution for 15 min. Infected poplar leaves, stem segments and tobacco leaves were placed in poplar co-cultivation medium (MS, 30 g L^−1^ sucrose, 6 g L^−1^ agar, 0.05 mg L^−1^ NAA and 0.5 mg L^−1^ 6-BA) or tobacco co-culture medium (MS, 30 g L^−1^ sucrose, 6 g L^−1^ agar, 0.01 mg L^−1^ NAA and 0.2 mg L^−1^ 6-BA) for 2 days in the dark at 25 ± 2 °C, and then transferred to poplar selective differentiation medium [MS, 30 g L^−1^ sucrose, 6 g L^−1^ agar, 0.05 mg L^−1^ NAA and 0.5 mg L^−1^ 6-BA, 30 mg L^−1^ Kan, and 200 mg L^−1^ Timentin (Tim)] or tobacco selective differentiation medium (MS, 30 g L^−1^ sucrose, 6 g L^−1^ agar, 0.01 mg L^−1^ NAA and 0.2 mg L^−1^ 6-BA, 30 mg L^−1^ Kan and 200 mg L^−1^ Tim) until resistant buds grew, and the regenerated shoot rate was counted. Finally, it was transferred to poplar selective rooting medium (1/2 MS, 30 g L^−1^ sucrose, 6 g L^−1^ agar, 0.02 mg L^−1^ NAA, 0.05 mg L^−1^ IBA, 30 mg L^−1^ Kan and 200 mg L^−1^ Tim) or tobacco selective rooting medium (MS, 30 g L^−1^ sucrose, 6 g L^−1^ agar, 0.4 mg L^−1^ IBA, 30 mg L^−1^ Kan and 200 mg L^−1^ Tim), and the rooting rate was counted. The proportion of positive seedlings was counted after PCR detection, and the primers used for PCR are shown in Additional file 6: Table S1.

### Statistical analysis

Statistical analyses were conducted using SPSS software (IBM Corp., Armonk, NY, USA). An analysis of variance was performed, and Duncan’s multiple range test was used to assess the differences between treatments. *P* < 0.05 was considered significant.

## Results

### Characteristics of morphological changes in plant in vitro culture materials

**Fig. 1:**
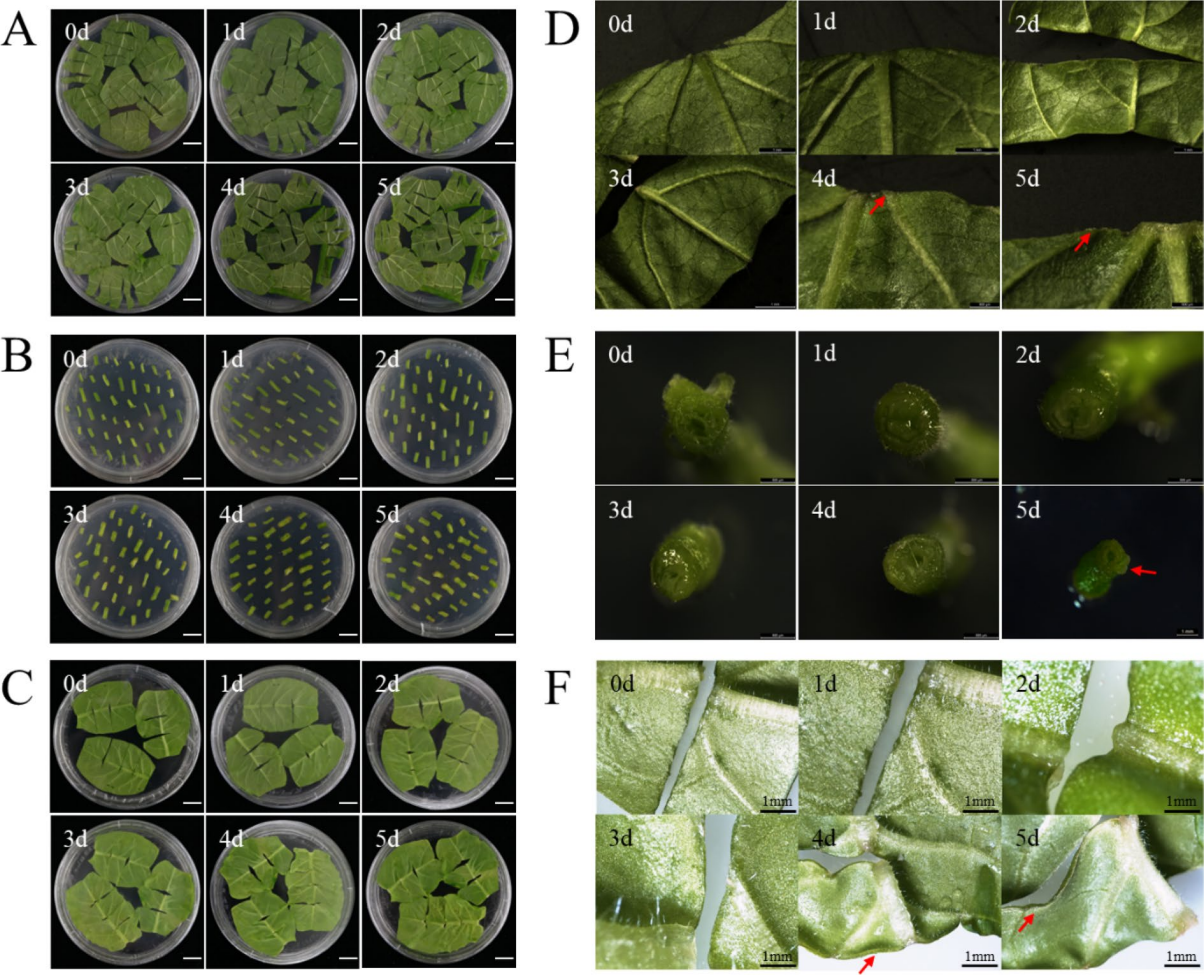
Phenotypic characteristics of poplar and tobacco under different culture times in vitro. Phenotypic characteristics of poplar leaves **A**, poplar stem segments **B**, and tobacco leaves **C** were cultured on plates containing differentiation medium for 0–5 days. Bars = 1 cm. Poplar leaves **D**, poplar stem segments **E**, and tobacco leaves **F** were observed under a stereo microscope after 0–5 days of differentiation culture. The red arrow marked the place where healing tissue was produced

The morphological changes of poplar leaves and stem segments and tobacco leaves cultured in vitro on differentiation medium at different times were observed. With the increase in culture time, the leaves gradually curled, and the stem segments gradually expanded (Fig. 1A–C). The incision of in vitro poplar leaves cultured on differentiation medium for 1 day was moist and green, and the leaves were flat without shrinkage. The poplar leaves that were cultured for 2 days became dry and wrinkled at the incision. Whole poplar leaves curled when cultured for 3 days. A small amount of callus formation was observed after 4 days of culture. After 5 days of culture, a small amount of callus began to appear on the entire incision, and the callus was white and soft (Fig. 1D).

For the in vitro culture of poplar stem segments, the epidermis cultured on differentiation medium for 1 and 2 days was dark green, and the cut surface was moist. When cultured for 3 days, the epidermis of the isolated stem segment was dark green, the incision sections at both ends became dry, and the edges began to swell. When cultured for 4 days, the epidermis of the stem segment was green, the incision sections at both ends began to lose water and shrink, and the outer edge swelled. After 5 days of culture, the epidermis of the stem segment was light green, the incisions at both ends were significantly enlarged, and a small number of callus appeared. The callus was white and small (Fig. 1E).

As far as the in vitro culture of tobacco leaves is concerned, the incision of tobacco leaves cultivated for 1 day was smooth, and the leaves were flat. The incision of the tobacco leaves that had been cultivated for 2 days began to curl, and the incision became dry. There was a slight bulge at the incision of the leaf when cultured for 3 days. When cultured for 4 days, the incision of the leaf shrank and lost water, and the color of the edge of the leaf turned yellow; the lower epidermis was slightly curled, and there was a very small amount of pale-yellow callus protrusions at the main vein of the leaf. After 5 days of culture, the incision of the leaf showed dehydration and shrinkage, the edge of the leaf was obviously curled, and a white callus had formed at the incision of the leaf, but it did not swell and became granular (Fig. 1F).

### Cytological observation of the developmental state of in vitro cultured plant materials

**Fig. 2:**
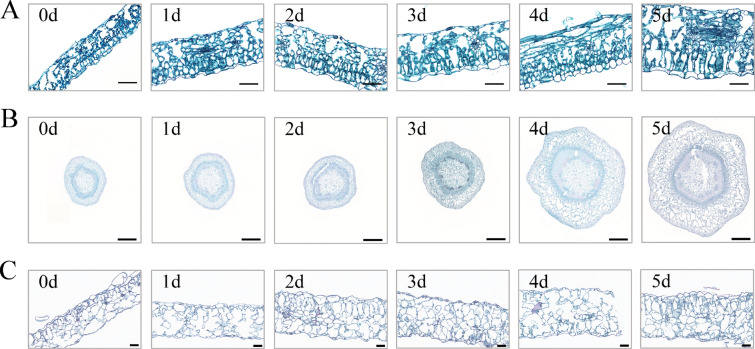
Observation of the cell development status of poplar and tobacco in vitro culture materials. **A** Cell development status of poplar leaves at 0, 1, 2, 3, 4, and 5 days of differentiation culture. Bars = 50 μm. **B** Cell development status of poplar stem segments at 0, 1, 2, 3, 4, and 5 days of differentiation culture. Bars = 500 μm. **C** Cell development status of tobacco leaves at 0, 1, 2, 3, 4, and 5 days of differentiation culture. Bars = 50 μm

Microscopic observation of paraffin sections was conducted on the incisions of poplar leaves and stem segments and tobacco leaves cultured in vitro on a differentiation medium, and there were differences in the occurrence and development of leaf bud primordium cells. Among them, when poplar leaves were cultured on differentiation medium for 1–2 days, compared with the control, the leaf epidermal cells gradually became larger, and the palisade tissue was still oval and arranged neatly; the size and shape of the spongy tissue were irregular; a small number of small cells with no vacuoles and dense cytoplasm were seen, and their nuclei were gradually stained (Fig. 2A). After culturing on differentiation medium for 3 days, more small cells with dense cytoplasm were observed, and their nuclei were enlarged and darkened, indicating that some leaf bud primordium cells were activated, and small cell clusters were clearly formed (Fig. 2A; Additional file 1: Fig. S1A). When poplar leaves were cultured for 4–5 days, the epidermal cells were elongated on both sides from oval to rod-shaped; palisade tissue and spongy tissue were closely arranged, and a small number of small cells with dense cytoplasm could be seen. At this time, the differentiation of leaf bud primordium cells was obvious (Fig. 2A). As the differentiation culture time increased, the leaves became larger and the thickness of mesophyll cells increased, especially after day 3, and the thickness of mesophyll cells reached its maximum on day 5 (Fig. 2A).

We observed the incision of the stem section of poplar and found that after 1–2 days of differentiation culture at the incision, nucleus staining was not obvious, and the difference in cell size was not significant (Fig. 2B). After poplar stems were cultured for 3 days, a small number of small cells without vacuoles and dense cytoplasm were observed, the nuclei were stained, and the cell cross-section was slightly enlarged (Fig. 2B). After being cultured for 4 days, the cross-section became significantly larger, the size of the cells at the incision increased significantly, and the edges were irregular in shape. Many small cells without vacuoles and dense cytoplasm were observed, and their nuclei were enlarged and darkened, indicating that budding primordium cells were activated. Significantly more cell clumps were observed at the cambium, whereas no or very few cell clumps were observed at the incision of the stem segment in the undifferentiated culture (0 days) (Fig. 2B; Additional file 1: Fig. S1B). After poplar stem segments were cultured for 5 days, the cell cross-section reached the maximum, the cell size was evenly distributed, and several small cells with dense cytoplasm could be seen. The leaf bud primordium cells entered the division and differentiation stages (Fig. 2B).

After differentiation culture of tobacco leaves for 1 day, the intercellular space at the incision was larger, and the staining of nuclei was not obvious (Fig. 2C). After being cultured for 2 days in tobacco leaves, we observed that leaf epidermal cells began to expand, the intercellular space became smaller, and many small cells without vacuoles and dense cytoplasm were seen (Additional file 1: Fig. S1C). The nuclei were clearly stained, and significantly more cell clusters were observed, while no cell clusters or very few cell clusters were seen in the undifferentiated tobacco leaves (0 days) (Fig. 2C). After differentiation culture of tobacco leaves for 3 days, palisade tissue and spongy tissue were irregular in size and shape, and a small number of small cell clusters with no vacuoles and dense cytoplasm were seen (Fig. 2C). After differentiation culture of tobacco leaves for 4–5 days, several small cells with dense cytoplasm were observed, and the differentiation of leaf bud primordium cells was obvious (Fig. 2C).

### Identification of the cell cycle of plant receptor materials

**Fig. 3:**
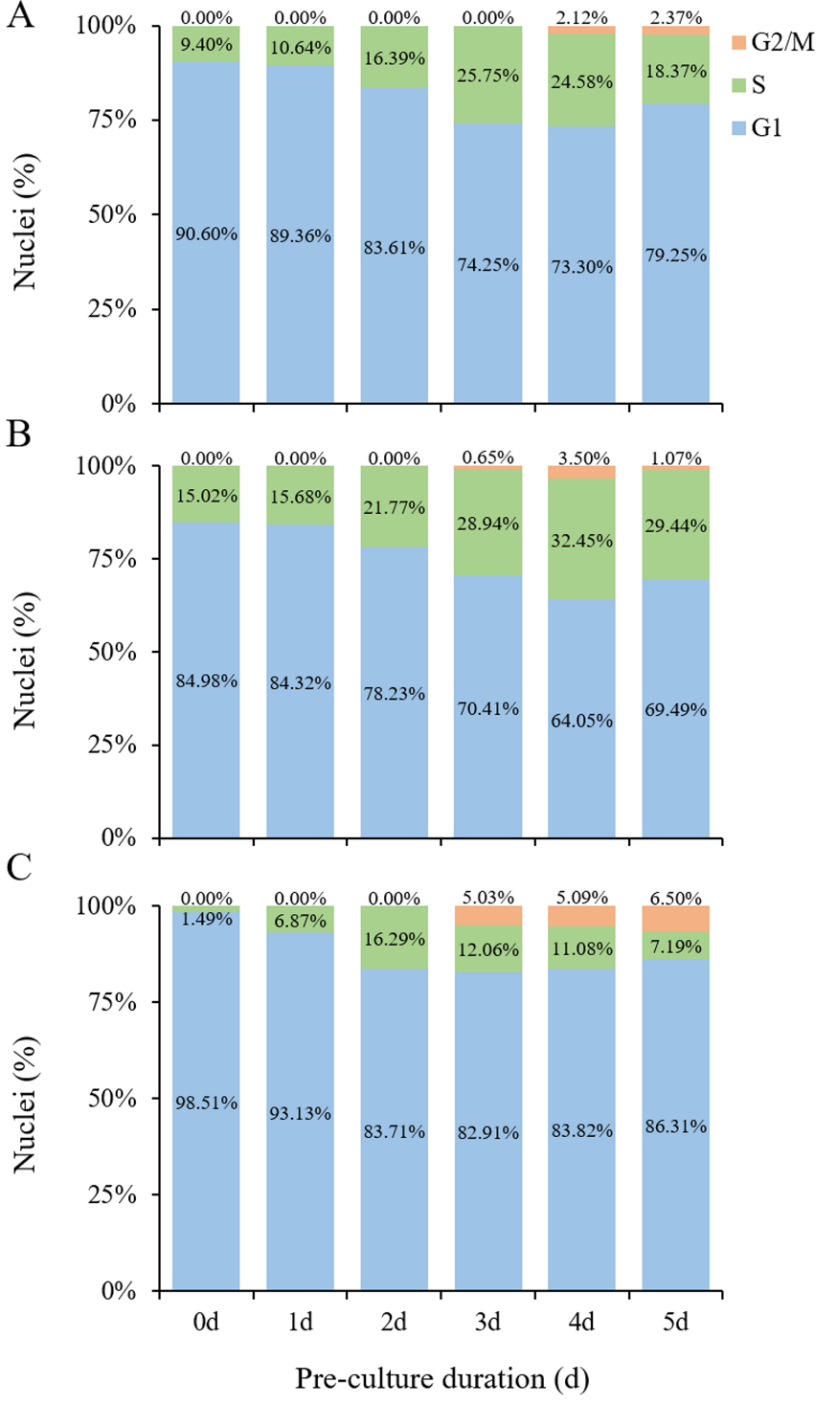
The cell cycle of the receptor material was detected by flow cytometry. **A** The percentage of cell cycle G1, S and G2/M phases of leaves of 84 K poplar after different differentiation culture time. **B** The percentage of cell cycle G1, S and G2/M phases of stem segments of 84 K poplar after different differentiation culture time. **C** The percentage of cell cycle G1, S and G2/M phases of tobacco leaves after different differentiation culture time

The mitotic cell cycle refers to the whole process that a cell undergoes, from the completion of one division to the completion of the next division. It is divided into two key stages: interphase and mitosis. Interphase includes the G1, S, and G2 phases [53]. Flow cytometry was used to detect the leaves of poplar cultured on differentiation medium at different times in vitro. With the extension of culture time, the proportion of cells in the G1 phase gradually decreased from 90.60% of the control, while the proportion of cells in the S phase gradually increased from 9.4%. The S phase cells reached a peak value on the 3rd day of differentiation culture, accounting for 25.75%, while the G1 phase cells decreased to 74.25%. The G2/M phase cells could not be detected until the 4th day of differentiation culture, accounting for 2.12%. On the 5th day of differentiation culture, cells in the G1 phase accounted for 79.25%, cells in the S phase accounted for 18.37%, and cells in G2/M phase accounted for 2.37% (Fig. 3A; Additional file 2: Fig. S2A). The flow cytometry results of poplar stem segments showed that the proportion of G1 and S phase cells in undifferentiated stem segments was 84.98% and 15.02%, respectively, and G2/M phase cells could not be detected. With the increase in differentiation culture time, the proportion of S phase cells increased and reached its peak on the 4th day. At this time, the proportion of S phase cells reached 32.45%, and the proportion of G2/M phase cells reached 3.5%. On the 5th day, the proportion of cells in the S and G2/M phases decreased again, with 29.44% in the S phase and 1.07% in G2/M phase (Fig. 3B; Additional file 2: Fig. S2B). The flow cytometry results of tobacco leaves showed that the proportion of S phase cells increased after 0–2 days of differentiation culture and reached the highest level on the 2nd day, accounting for 16.29% at this time, but no G2/M phase cells were detected. After the 3rd day, the proportion of cells in the S phase began to decrease again. On the 5th day, the proportion of cells in the S phase was 7.19%, while that in G2/M phase was the highest, 6.50% (Fig. 3C; Additional file 2: Fig. S2C).

**Fig. 4:**
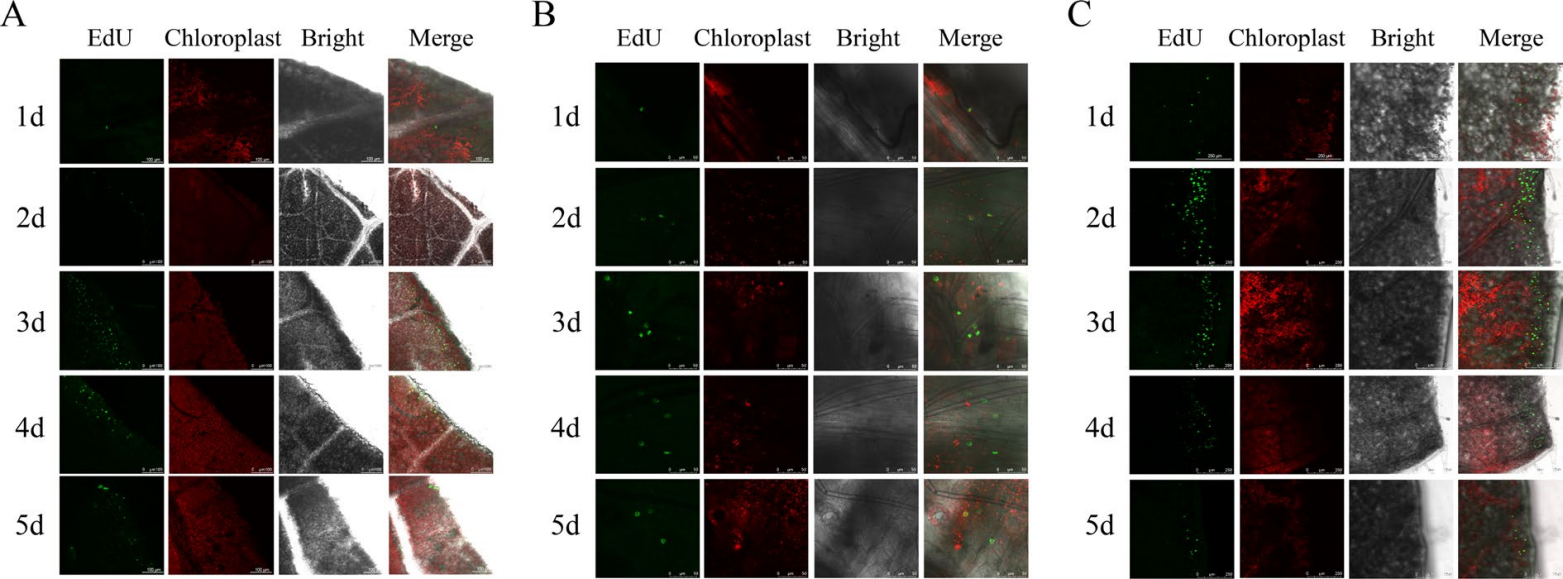
EdU staining was used to identify the number of cells in the S phase of the cell cycle. The EdU fluorescence, chloroplast, bright field, and merge signal of poplar leaves **A**, poplar stem segments **B**, and tobacco leaves **C** after differentiation culture for 1, 2, 3, 4, and 5 days were observed under a confocal microscope

EdU staining can accurately locate cells in the S phase of the cell cycle and make them emit green fluorescence [54]. We used this method to stain poplar and tobacco in vitro at different times and observed their fluorescence signal distribution. Only a small number of S phase cells emitting green fluorescence were observed in the leaves of poplar after 1 day of differentiation culture. On the 2nd day of differentiation culture, more S phase cells began to appear (Fig. 4A). On the 3rd day of differentiation culture, many S phase cells appeared in the leaves of poplar, which was in the DNA replication phase. At this time, the number of S phase cells reached a peak, and they were evenly distributed at the leaf incision (Fig. 4A). On the 4th day of the differentiation culture of poplar leaves, the number of cells in the S phase began to decrease, suggesting that many cells began to enter the G2 phase or division phase (Fig. 4A). On the 5th day of differentiation culture of poplar leaves, the number of cells in the S phase was significantly reduced and scattered at the edge of the incision (Fig. 4A). On the 6th day of differentiation culture of poplar leaves, transparent calli were obviously visible at the incision. At this time, fewer cells were in the S phase of the cell cycle, significantly less than the number of cells on the 5th day (Additional file 3: Fig. S3). The EdU staining results of the stem segments of poplar showed that after 1 day of differentiation culture, cells in the S phase of the cell cycle began to appear, and gradually increased from the 1st to the 4th day, reaching the maximum on the 4th day. However, on the 5th day, the number of S phase cells decreased (Fig. 4B). Similarly, our EdU staining results on tobacco leaves showed that S phase cells appeared after the 1st day of differentiation culture, and many S phase cells appeared on the 2nd day, reaching a peak. From the 3rd to the 5th day, the number of S phase cells emitting green fluorescence gradually decreased (Fig. 4C).

### Changes in cyclin gene differential expression in receptor materials in different developmental states of plants

**Fig. 5:**
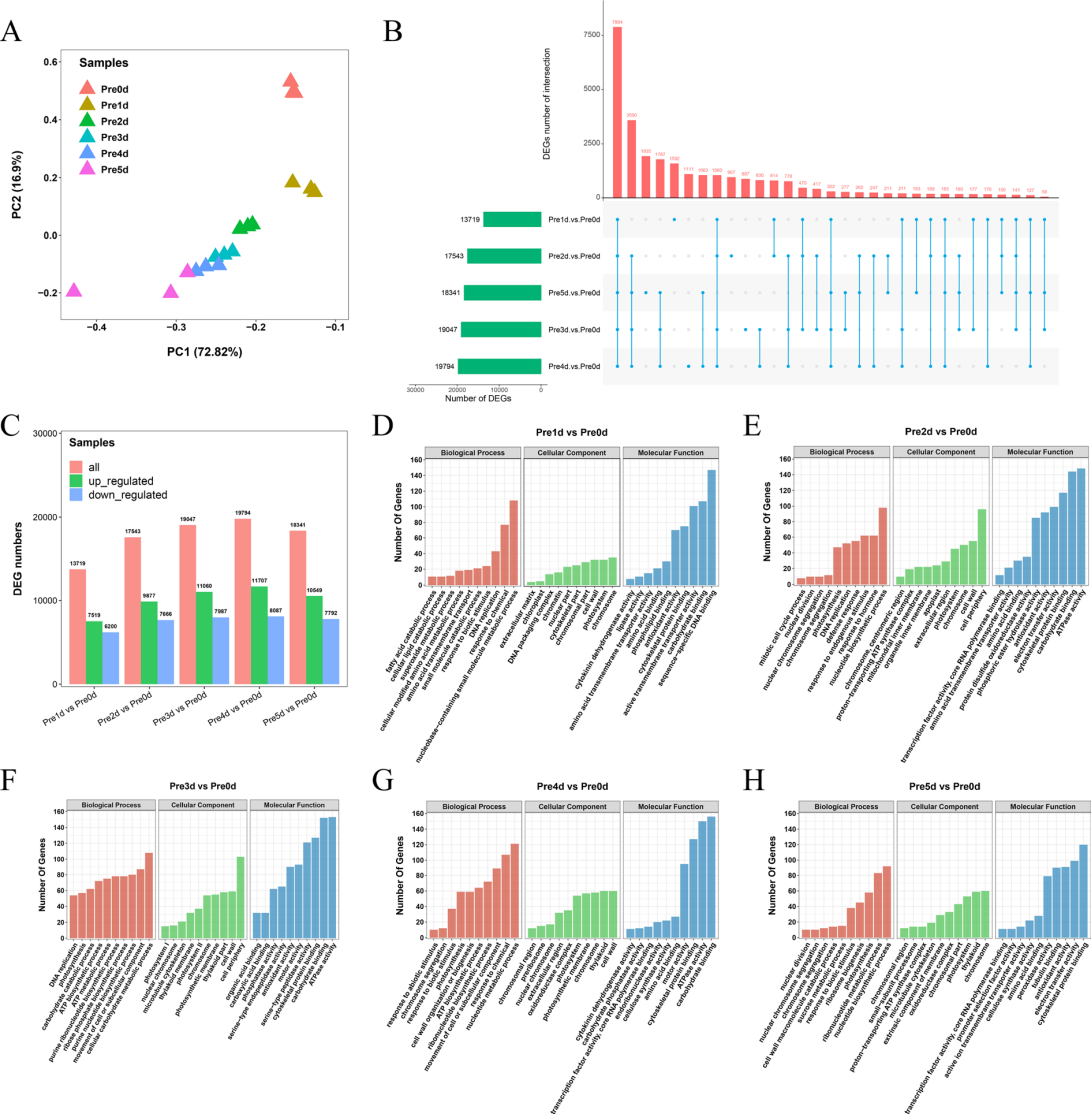
Analysis of transcriptome sequencing data. **A** Principal component analysis (PCA) analysis of transcriptome sequencing samples. **B** Upset plot of differentially expressed gene (DEGs) in different culture durations. The top column represents the DEGs’ number of intersections, and the left column graph represents the DEGs’ number of each dataset. Five separate blue dots represent the DEGs that are only present in one dataset. The DEGs present in at least two datasets are marked as connected blue dots. The connection represents the intersection, and the blue dots on the line represent the corresponding datasets. **C** The number of differentially expressed upregulated and downregulated genes between cultured and not cultured. **D** Gene Ontology (GO) enrichment analysis of DEGs between differentiation cultures at 1 and 0 days. **E** GO enrichment analysis of DEGs between differentiation cultures at 2 and 0 days. **F** GO enrichment analysis of DEGs between differentiation cultures at 3 and 0 days. **G** GO enrichment analysis of DEGs between differentiation cultures at 4 and 0 days. **H** GO enrichment analysis of DEGs between differentiation cultures at 5 and 0 days

Transcriptome sequencing of poplar leaves cultured in vitro at different times on differentiation medium was performed. Principal component analysis (PCA) results showed that there was a high degree of similarity between the biological replicates of each sample, indicating that the sequencing data were relatively reliable and suitable for further analysis (Fig. 5A; Additional file 6: Table S2). Compared with the control, the DEG numbers of poplar leaves cultured in vitro on differentiation medium for 1, 2, 3, 4, and 5 days were 13,719, 17,543, 19,047, 19,794, and 18,341, respectively (Fig. 5B and C) There were 7884 DEGs in common (Fig. 5B; Additional file 6: Tables S3 and S4). Compared with undifferentiated poplar leaves, three pathways that might be related to S phase, DNA replication, chromosome, and carbohydrate binding pathways were significantly enriched at 1–3 days after differentiation culture (Fig. 5D–F; Additional file 6: Tables S5–S7). Chromosome segregation, chromosome, and cytoskeletal protein binding pathways were significantly enriched at 4–5 days of differentiation culture (Fig. 5G and H; Additional file 6: Tables S8 and S9).

**Fig. 6:**
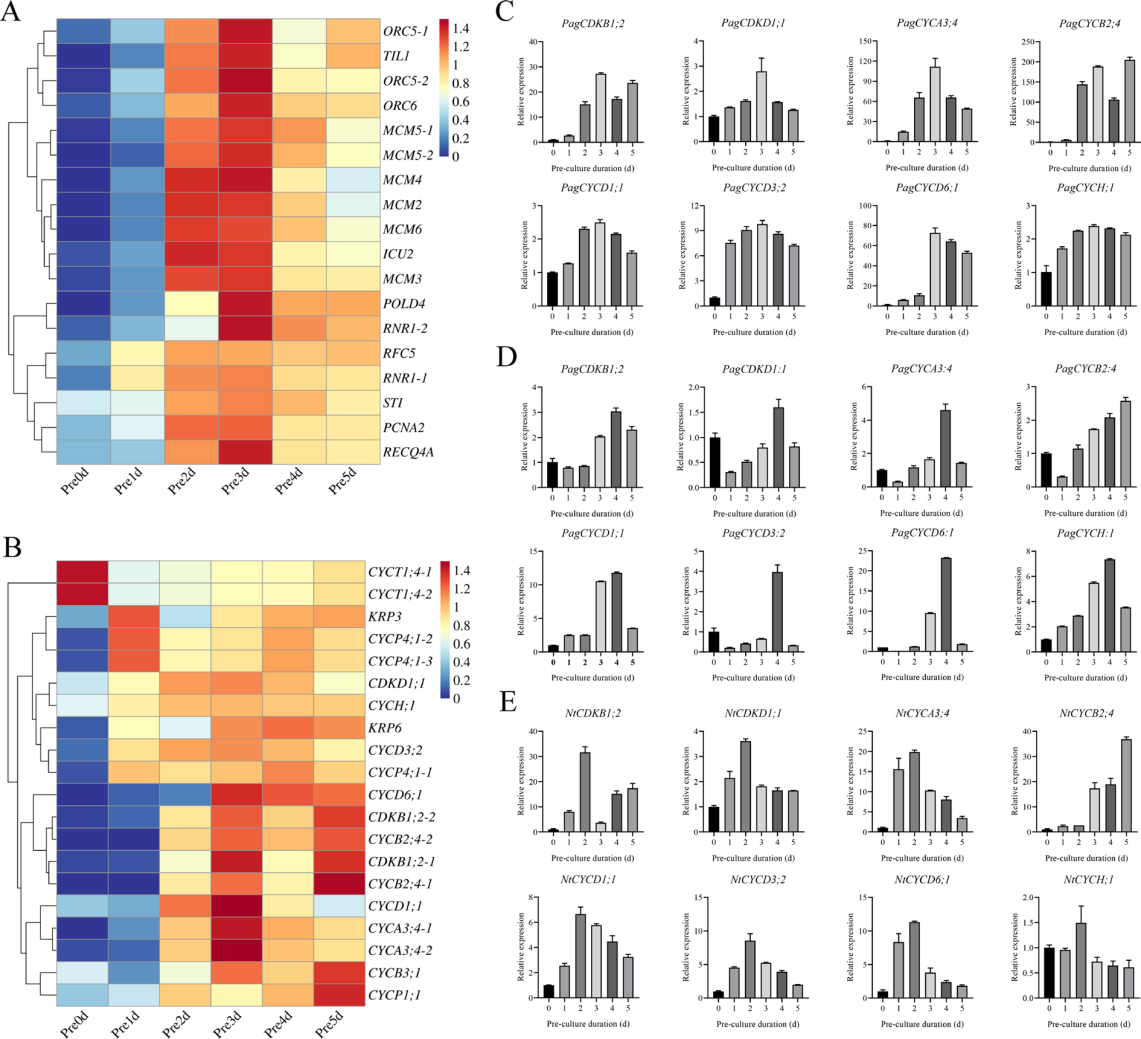
Expression levels of genes related to DNA replication and the cell cycle. **A** Changes in the expression levels of DNA replication-related genes at 0–5 days of differentiation culture. **B** Changes in the expression levels of cell cycle-related genes at 0–5 days of differentiation culture. **C** RT-qPCR detection of the expression of cell cycle-related genes (*PagCDKB1;2*, *PagCDKD1;1*, *PagCYCA3;4*, *PagCYCB2;4*, *PagCYCD1;1*, *PagCYCD3;2*, *PagCYCD6;1*, and *PagCYCH;1*) in poplar leaves in differentiated cultured for 0–5 days. **D** RT-qPCR detection of the expression of cell cycle-related genes (*PagCDKB1;2*, *PagCDKD1;1*, *PagCYCA3;4*, *PagCYCB2;4*, *PagCYCD1;1*, *PagCYCD3;2*, *PagCYCD6;1*, and *PagCYCH;1*) in poplar stem segments in differentiated cultured for 0–5 days. Expression was estimated by RT-qPCR normalized to *PagACTIN* expression. **E** RT-qPCR detection of the expression of cell cycle-related genes (*NtCDKB1;2*, *NtCDKD1;1*, *NtCYCA3;4*, *NtCYCB2;4*, *NtCYCD1;1*, *NtCYCD3;2*, *NtCYCD6;1*, and *NtCYCH;1*) in tobacco leaves in differentiated cultured for 0–5 days. Expression was estimated based on RT-qPCR normalized to *NtACTIN* expression. Error bars represent the SD (data are the means of the three biological replicates). *P* < 0.05 is considered significantly different and is shown by different letters

Because the DNA replication pathway was significantly enriched in poplar leaves after differentiation culture, we further analyzed the expression trends of its related genes. The expression trends of some DNA replication-related genes were relatively consistent under different differentiation culture time treatments, such as *ORC5*, *TIL1*, *ORC6*, *MCM5*, *MCM4*, *MCM3*, *POLD4*, *RNR1*, *STI*, and *RECQ4A*. Their expression level was the highest in the leaves at day 3, and the expression level was generally lower at day 0 of the differentiation culture. From day 0 to 5 of differentiation culture, there was an expression trend similar to a normal distribution (Fig. 6A; Additional file 6: Table S10). We further analyzed the differential expression changes of G1, S, and G2 phase-related genes in the leaves of poplars cultured in vitro for 1, 2, 3, 4, and 5 days and found 20 genes in total. Among them, *CYCT1;4* was significantly highly expressed in the G1 phase, and its expression in poplar leaves gradually decreased with an increase in differentiation culture time. It is speculated that *CYCT1;4* mainly plays a role in the G1 phase. The expression levels of cyclins *CDKB1;2*, *CDKD1;1*, *CYCA3;4*, *CYCD1;1*, *CYCD3;2*, *CYCD6;1*, and *CYCH;1* reached the highest level on the 3rd day of differentiation culture (Fig. 6B; Additional file 6: Table S11). The expression levels of cyclins *CYCB2;4*, *CYCB3;1*, and *CYCP1;1*, which function in the G2/M phase of the cell cycle, were highest in the leaves on the 5th day of differentiation culture (Fig. 6B; Additional file 6: Table S11).

To verify the results of transcriptome sequencing, we selected the expression levels of eight genes at different differentiation culture times for RT-qPCR detection. When poplar leaves were cultured for 0–5 days, the expressions of cyclin kinases *PagCDKB1;2* and *PagCDKD1;1* and cyclins *PagCYCA3;4*, *PagCYCD1;1*, *PagCYCD3;2*, *PagCYCD6;1*, and *PagCYCH;1* that function in the S phase first increased and then decreased, and reached a peak on day 3 (Fig. 6C). However, the expression of *PagCYCB2;4*, which functions in the G2/M phase, reached the highest value in the poplar leaves on day 5 of the differentiation culture (Fig. 6C).

Subsequently, we detected the expression levels of the above eight genes after differentiation culture of poplar stem segments for 0–5 days. Similar to poplar leaves, *PagCDKB1;2*, *PagCDKD1;1*, *PagCYCA3;4*, *PagCYCD1;1*, *PagCYCD3;2*, *PagCYCD6;1*, and *PagCYCH;1* were expressed at the highest level in the S phase of the cell cycle, that is, on the 4th day of differentiation culture (Fig. 6D). However, *PagCYCB2;4*, which functions in the G2/M phase, showed an increasing trend from the 1st to the 5th day of differentiation culture, and the highest expression level was found in the poplar stem segments on the 5th day of differentiation culture (Fig. 6D).

We also detected the expression of *NtCDKB1;2*, *NtCDKD1;1*, *NtCYCA3;4*, *NtCYCB2;4*, *NtCYCD1;1*, *NtCYCD3;2*, *NtCYCD6;1*, and *NtCYCH;1* in tobacco leaves after 0–5 days of differentiation culture and found that they also had different expression patterns under different differentiation culture times. *NtCDKB1;2*, *NtCDKD1;1*, *NtCYCA3;4*, *NtCYCD1;1*, *NtCYCD3;2*, *NtCYCD6;1*, and *NtCYCH;1*, which function in the S phase, were all expressed at the highest level on day 2 of differentiation culture. However, *NtCYCB2;4* expression, which plays a role in the G2/M phase, showed an upward trend with the increase in differentiation culture time and reached a peak on the 5th day of differentiation culture (Fig. 6E).

### Genetic transformation efficiency of plant receptor materials under different cell development states

**Fig. 7:**
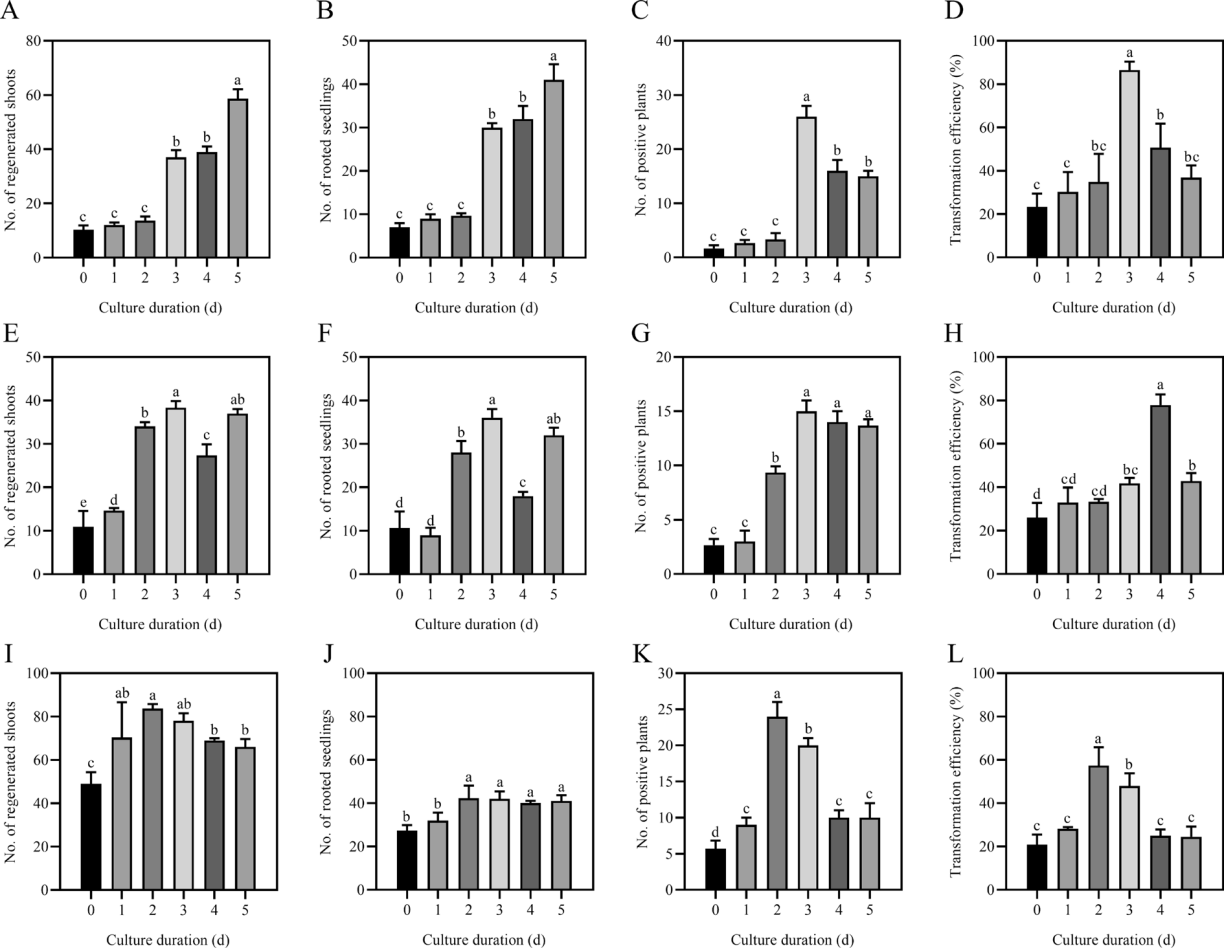
Genetic transformation efficiency of in vitro materials at different differentiation culture times. **A** The number of regenerated shoots after differentiation culture of poplar leaves; **B** the number of rooted seedlings after differentiation culture of poplar leaves; **C** the number of positive plants after differentiation culture of poplar leaves; **D** transformation efficiency after differentiation culture of poplar leaves; **E** the number of regenerated shoots after differentiation culture of poplar stem segments; **F** The number of rooted seedlings after differentiation culture of poplar stem segments; **G** the number of positive plants after differentiation culture of poplar stem segments; **H** transformation efficiency after differentiation culture of poplar stem segments; **I** the number of regenerated shoots after differentiation culture of tobacco leaves; **J** the number of rooted seedlings after differentiation culture of tobacco leaves; **K** the number of positive plants after differentiation culture of tobacco leaves; **L** transformation efficiency after differentiation culture of tobacco leaves. The data presented are the mean ± standard deviation (SD) of three replicates. Bars with different letters are significantly different based on one-way ANOVA analysis (P < 0.05)

Differentiation culture of the recipient material can induce callus formation for better budding [55]. To study the genetic transformation efficiency of different plant acceptor materials under different cell development states, we used poplar leaves, poplar stem segments and tobacco leaves as acceptor materials. After the recipient material was cultured for 0, 1, 2, 3, 4, and 5 days, it was infected with *Agrobacterium*, and after the recipient material regenerated on the selective differentiation medium, it was transferred to the selective rooting medium for rooting (Additional file 4: Fig. S4). DNA extraction and positive seedling detection were performed on the root tissue culture seedlings. Those with bands detected using agarose gel electrophoresis were positive seedlings and positive controls, and those without bands were negative controls (Additional file 5: Fig. S5). Without differentiation culture treatment, the genetic transformation efficiency of poplar leaves, poplar stem segments and tobacco leaves was about 23.4% (Fig. 7D), 26.1% (Fig. 7H), and 20.9% (Fig. 7L).

After the poplar leaves were cultured, the number of regenerated shoots, rooted seedlings, positive plants, and the transformation efficiency at 3, 4, and 5 days were higher than at 0 days of differentiation culture (Fig. 7A–D). Among them, the transformation efficiency of poplar leaves was the highest when they were cultured for 3 days, reaching 86.6% (Fig. 7D).

After the poplar stem segments were cultured, the number of regenerated shoots, rooted seedlings, positive plants, and the transformation efficiency at 2, 3, 4, and 5 days were higher than at 0 days of differentiation culture (Fig. 7E–H). The transformation efficiency of poplar stem segments was highest when cultured for 4 days, reaching 77.8% (Fig. 7H). The number of regenerated shoots, positive plants, and the transformation efficiency after 1 day of differentiation culture were higher than after 0 days (Fig. 7E, G and H), but the rooted seedlings were lower than after 0 days (Fig. 7F).

After differentiation culture of tobacco leaves, the number of regenerated shoots at 1–5 days was significantly higher than that at 0 days (Fig. 7I), and the rooted seedlings at 2, 3, 4, and 5 days of differentiation culture were not significantly different and slightly higher at 0 and 1 day (Fig. 7J). The number of positive plants and transformation efficiency at 1–5 days of differentiation culture were higher than those without differentiation culture (Fig. 7K and L), and the transformation efficiency was the highest at 2 days after differentiation culture, reaching 57.3% (Fig. 7L). These results showed that the differentiation culture of in vitro materials before genetic transformation treatment significantly improved the efficiency of plant genetic transformation. Among them, poplar leaves generally had the highest transformation efficiency when they were cultured for 3 days, poplar stem segments had the highest transformation efficiency when they were cultured for 4 days, and tobacco leaves had the highest transformation efficiency when they were cultured for 2 days.

## Discussion

Plant genetic transformation is very important for basic biology research and modern plant breeding. At present, the *Agrobacterium*-mediated genetic transformation method is a widely used transformation technology [1]. *Agrobacterium tumefaciens* is a gram-negative soil bacterium that contains the Ti plasmid. T-DNA is located on the Ti plasmid, with 25 bp repeats (LB and RB) on both sides [56]. These sequences are the recognition sites of virulence proteins VirD1 and VirD2. They produce single-stranded DNA (ssDNA) breaks and release T-DNA into ssDNA molecules, namely the T-strand [56]. *Agrobacterium tumefaciens* attaches to the injured plant, transfers its T-DNA fragments, and integrates them into the genome of the recipient plant [57]. There are two ways to integrate T-DNA transfer into the recipient genome: homologous recombination (HR), which uses homology between fragments to replace the target gene of the recipient plant, and abnormal recombination [terminal junction (EJ)], that is, random integration of T-DNA into recipient plant genomes [37, 58]. Because T-DNA does not need to be homologous with the target integration site, EJ has always been the preferred mechanism for T-DNA integration [58–60]. Homologous recombination often occurs in the S phase of the cell cycle, while the EJ pathway plays a role throughout interphase and is inhibited during mitosis [53]. The EJ pathway is subdivided into the non-homologous terminal junction pathway (NHEJ) and the terminal junction pathway (TMEJ). NHEJ repairs DSB in G1 and S phase pre-replication DNA [53], while TMEJ occurs in the late phase of the cell cycle, S/G2/M phase [61]. Therefore, the cell cycle phase of host cells during infection may determine the path selection and *Agrobacterium*-mediated genetic transformation results [62]. The process of T-DNA integration into the plant genome depends heavily on the TMEJ pathway [63], and transformation takes place in S phase cells [64].

It is generally believed that the regeneration of receptor materials, such as plant leaves and stem segments, is mainly caused by the dedifferentiation and cell division of parenchyma cells, epidermal cells, and vascular bundle sheath cells around the incision and its vicinity to produce meristematic cell clusters [65–67]. Under in vitro culture conditions, there are differences in the development of adventitious bud primordial cells of different species, different genotypes of the same species, and different explants of the same genotype [47, 55, 68–71], indicating that there are differences in the time when different species and different tissue materials enter the optimal period of genetic transformation under different conditions of in vitro culture. From the comparison of genetic transformation receptor materials, such as poplar leaves, stem segments and tobacco leaves in vitro culture, there are differences in morphology, anatomy, and the development process of adventitious bud primordial cells of three kinds of explant materials at different development stages under in vitro culture conditions. In terms of morphology and anatomy, the leaves of poplar and tobacco began to curl on the 3rd and 2nd day of culture, respectively, and callus was formed at the incision on the 4th day of culture (Fig. 1D and F). However, on the 5th day of culture, the cut at both ends of the stem segments of poplar was significantly expanded, and a few calli appeared (Fig. 1E). After 3 days of leaf culture, 4 days of stem segment culture, and 2 days of tobacco leaf culture, many small cells with dense cytoplasm were observed, their nuclei were enlarged, and their colors deepened (Fig. 2). Using flow cytometry and EdU staining, the number of cells in the S phase of leaves and stem segments of poplar and tobacco leaves was the largest at 3, 4, and 2 days of culture, respectively (Figs. 3, 4, and Additional file 2: Fig. S2). Transcriptome data analysis and RT-qPCR showed that the expression of DNA replication-related genes in poplar leaves after 3 days of culture was higher than that in the other treatments and controls (Fig. 5A). When the leaves of poplar were cultured for 3 days, the stem segments of poplar were cultured for 4 days, and the leaves of tobacco were cultured for 2 days, cell cycle-related genes *CDKB1; 2*, *CDKD1; 1*, *CYCA3; 4*, *CYCD1; 1*, *CYCD3; 2*, *CYCD6; 1*, and *CYCH; 1* had significantly high expression, promoting cell cycle transition to S phase, and relatively low expression at other treatment times (Fig. 5B–E). There were differences in the development process of adventitious bud primordial cells in poplar leaves and stem segments and tobacco leaves in vitro. In the process of genetic transformation, we should master the differences in different species and different tissue receptor materials entering the S phase of the cell cycle and select the best time for genetic transformation according to the development process of the materials in vitro.

In the process of polyploid induction, selecting the best treatment period and applying physical and chemical treatment can significantly improve the polyploid induction rate [55, 72], and different tree species and materials have different effective treatment periods for chromosome doubling [47, 55, 69, 71]. Studies have shown that among the many factors that determine the efficiency of genetic transformation by the leaf disc method, plant species and genotypes within the same species may have greater influence [31–34, 73]. The use of different explants and explants at different development stages has a great impact on transformation efficiency [6, 7, 19, 35, 37–40]. This indicates that the optimal genetic transformation period must be different for different species, different genotypes of the same species, different explants of the same genotype, and receptor materials of the same explant at different developmental stages when transformation is applied. Through statistical analysis of the genetic transformation efficiency of five differentiation and development stages of poplar leaves and stem segments and tobacco leaves, the optimal period of genetic transformation treatment was determined; that is, the highest genetic transformation efficiency was 86.6, 77.8, and 57.3%, respectively, for poplar leaves infected and cultured with *A. tumefaciens* for 3 days, poplar stem segments cultured for 4 days, and tobacco leaves cultured for 2 days (Fig. 7D, H and L) At this time, the explant materials belong to the majority of adventitious bud primordial cells entering the DNA replication phase; that is, the proportion of the S phase of the cell cycle is relatively high. In other periods, the genetic transformation rate obtained by *A. tumefaciens* infection was relatively low, which should be related to the fact that the cell development deviated from the highly replicating phase of DNA, and the proportion of S phase cells was relatively small. Obtaining certain genetically transformed plants after 0 days of culture may be related to the transformation and regeneration of adventitious bud primordial cells that occurred earlier in the cells. One day after the explant material was cultured, there were more or less leaf bud primordial cells entering the S phase every day. If *A. tumefaciens* is kept active on the recipient plant explants, theoretically, all leaf bud primordial cells can be transformed. The problem is that *Agrobacterium* can proliferate rapidly in the culture medium. To avoid the influence of the proliferation of *A. tumefaciens* on the development of the receptor material, generally, the receptor material needs to be transferred to a medium containing appropriate antibacterial antibiotics, such as Tim, for culture 2 days after immersion in the *A. tumefaciens* culture solution [41, 74]. This also means that the leaf disc method can improve the genetic transformation effect by considering the best treatment period.

In this study, a stable and efficient plant genetic transformation system based on an appropriate treatment period was established by studying the development status of different genetic transformation receptor materials of poplar leaves, stem segments, and tobacco leaves under in vitro culture conditions, as well as the actual effect of genetic transformation. It has been proven that there are differences in the cell development of different explants of different plants’ in vitro culture materials. The best treatment period of genetic transformation was the S phase of the cell cycle, as determined using flow cytometry detection, the EdU staining experiment, and expression analysis of *CDKB1; 2*, *CDKD1; 1*, *CYCA3; 4*, *CYCD1; 1*, *CYCD3; 2*, *CYCD6; 1*, and *CYCH; 1* for the in vitro culture materials. In practical operations, the best treatment period can be determined by combining the in vitro culture time and morphological changes in explants. This study provides a set of simple and efficient methods to determine the optimal treatment period of genetic transformation using the leaf disc method and provides theoretical support for other species to establish an efficient and stable genetic transformation system.

## Conclusions

In this study, we proposed an efficient and stable genetic transformation system using 84 K poplar and tobacco as materials. After differentiation culture of poplar leaves, stem segments and tobacco leaves, genetic transformation treatment was applied. Through morphological and cytological observation, flow cytometry detection, EdU staining, and the expression of cell cycle-related genes, the optimal treatment period, namely cell cycle S phase, was determined. At this time, the genetic transformation efficiency was the highest.

## Supplementary Information

**Additional file 1:** **Figure S1**. Leaf bud primordium cells. (A) Leaf bud primordium cells of poplar leaves on day 3 of preculture. (B) Leaf bud primordium cells of poplar stem segments on day 4 of preculture. (C) Leaf bud primordium cells of tobacco leaves on day 2 of preculture.

**Additional file 2: Figure S2**. The percentage of G1, S and G2/M phases of the cell cycle of the receptor material was detected by flow cytometry. After the leaves of poplar (A), stem segments of poplar (B), and tobacco leaves (C) were cultured for 0, 1, 2, 3, 4, and 5 d, the percentage of cells in the G1, S, and G2/M phases was detected by flow cytometry.

**Additional file 3: Figure S3**. EdU staining of 84K poplar leaves after 6 days of differentiation culture. The picture shows the images under four channels of EdU, Chloroplast, Bright and Merge. The cells in S phase of cell cycle emit green fluorescence.

**Additional file 4: Figure S4.** Regeneration of different receptor materials after treatment with *Agrobacterium tumefaciens*. (A) Phenotypic characteristics of 84K poplar leaves cultured on differentiation medium, budding and rooting. (B) Phenotypic characteristics of 84K poplar stem segments cultured on differentiation medium, budding and rooting. (C) Phenotypic characteristics of tobacco leaves cultured on differentiation medium, budding and rooting.

**Additional file 5: Figure S5**. Detection of positive plantlets obtained from genetic transformation after differentiation culture of different receptor materials for different times. (A) PCR detection of genetic transformation positive seedlings of 84K poplar leaves, this testing experiment being repeated at least three times with similar results; 1: wild-type poplar; 2: plasmid; 3-24: transgenic plants. (B) PCR detection of genetic transformation positive seedlings of 84K poplar stem segments, this testing experiment being repeated at least three times with similar results; 1: wild-type poplar; 2: plasmid; 3-24: transgenic plants. (C) PCR detection of genetic transformation positive seedlings of tobacco leaves, this testing experiment being repeated at least three times with similar results; 1: wild-type tobacco; 2: plasmid; 3-13: transgenic plants.

**Additional file 6: Table S1. **The primers used in this study. **Table S2. **Gene expression levels in different samples. **Table S3. **DEGs between samples treated with different differentiation culture time and those without differentiation culture. **Table S4. **The number of DEGs after different differentiation culture time treatment and without differentiation culture treatment. **Table S5. **GO enrichment analysis data between samples of differentiation culture 1d and differentiation culture 0d. **Table S6. **GO enrichment analysis data between samples of differentiation culture 2d and differentiation culture 0d.** Table S7. **GO enrichment analysis data between samples of differentiation culture 3d and differentiation culture 0d.** Table S8. **GO enrichment analysis data between samples of differentiation culture 4d and differentiation culture 0d.** Table S9. **GO enrichment analysis data between samples of differentiation culture 5d and differentiation culture 0d. **Table S10. **Expression of DNA replication related genes in 0-5 days of differentiation culture. **Table S11. **Expression of cell cycle related genes in 0-5 days of differentiation culture.

## Data Availability

The datasets used in this study are available from the corresponding author on reasonable request.
