# Supplementary figures and images for: Effect of a suitable treatment period on the genetic transformation efficiency of the plant leaf disc method
Source: Plant Methods. 2023 Feb 15;19:15. doi: 10.1186/s13007-023-00994-3 (PMC9930321; doi:10.1186/s13007-023-00994-3)

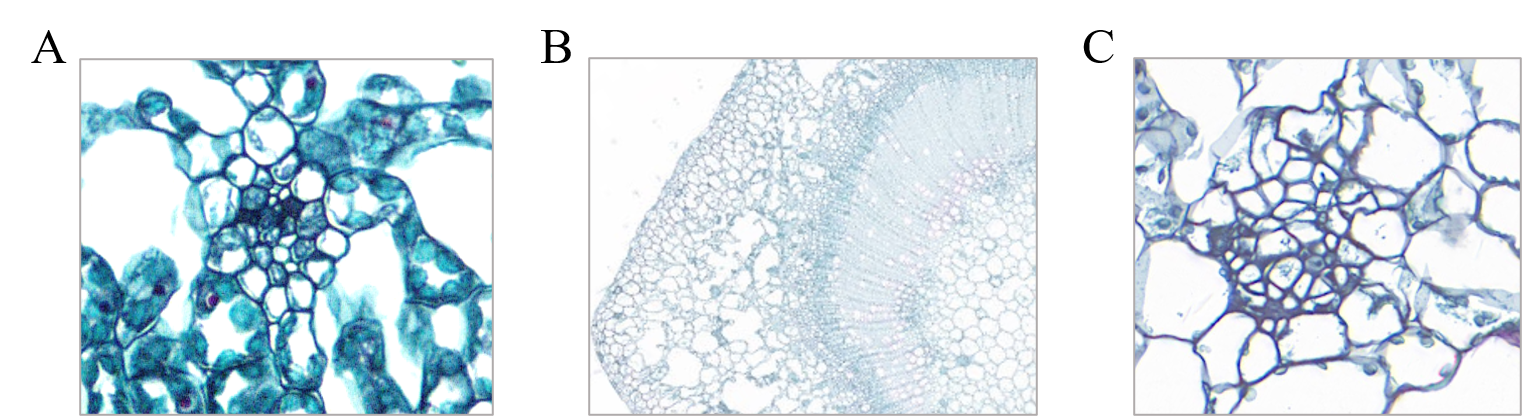

Supplement: Supplementary file 1 — Additional file 1: Figure S1. Leaf bud primordium cells. (A) Leaf bud primordium cells of poplar leaves on day 3 of preculture. (B) Leaf bud primordium cells of poplar stem segments on day 4 of preculture. (C) Leaf bud primordium cells of tobacco leaves on day 2 of preculture. [file 13007_2023_994_MOESM1_ESM.tif]

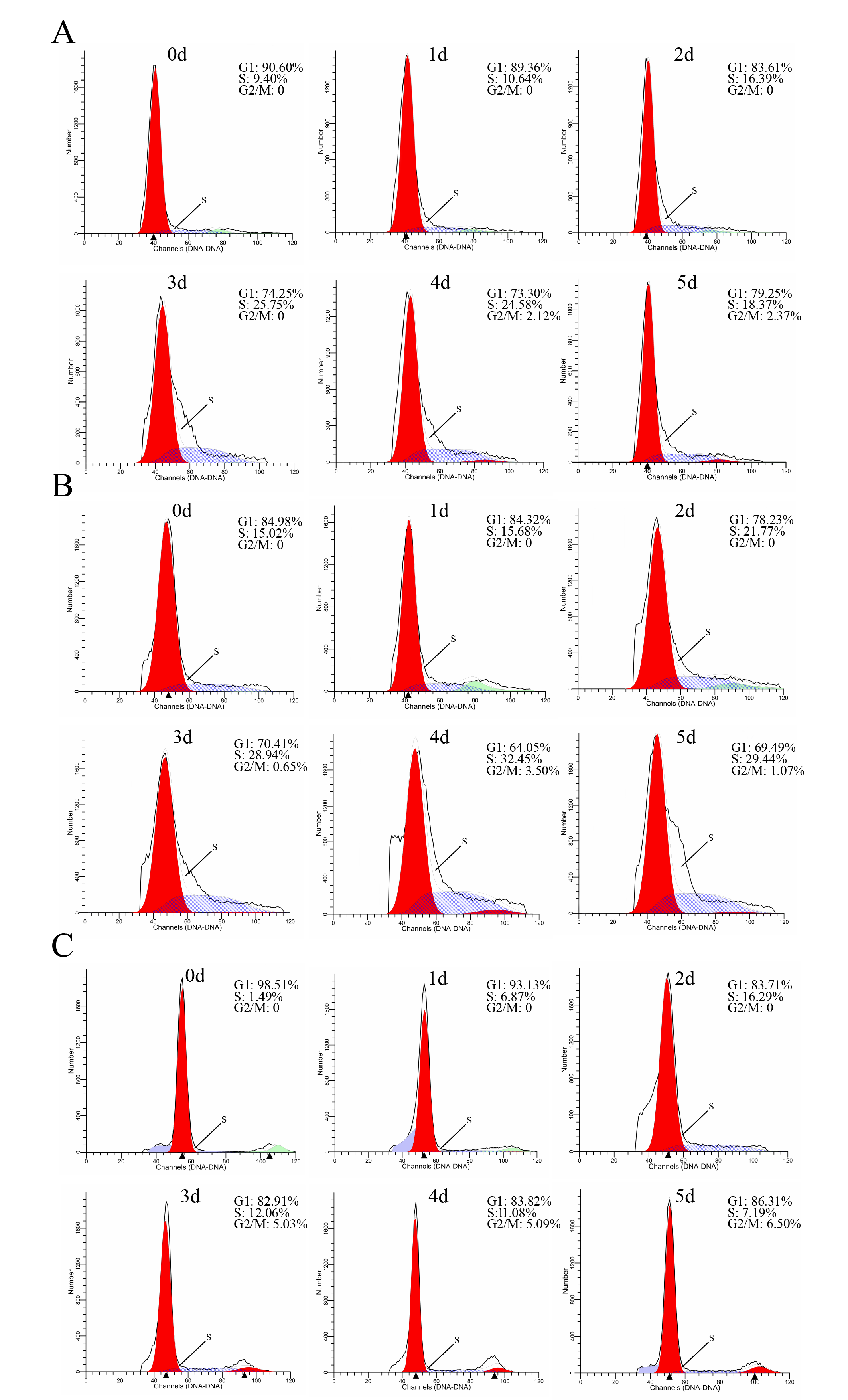

Supplement: Supplementary file 2 — Additional file 2: Figure S2. The percentage of G1, S and G2/M phases of the cell cycle of the receptor material was detected by flow cytometry. After the leaves of poplar (A), stem segments of poplar (B), and tobacco leaves (C) were cultured for 0, 1, 2, 3, 4, and 5 d, the percentage of cells in the G1, S, and G2/M phases was detected by flow cytometry. [file 13007_2023_994_MOESM2_ESM.png]

Pre6d

EdU

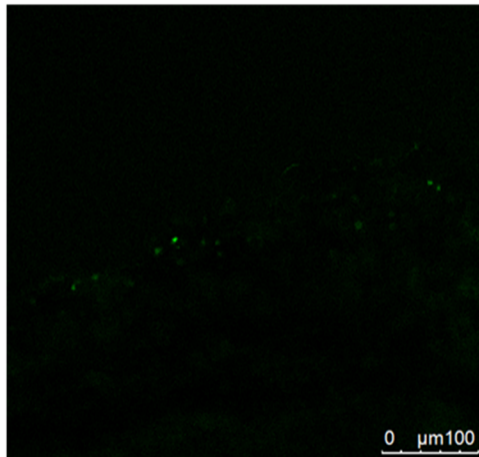

Chloroplast

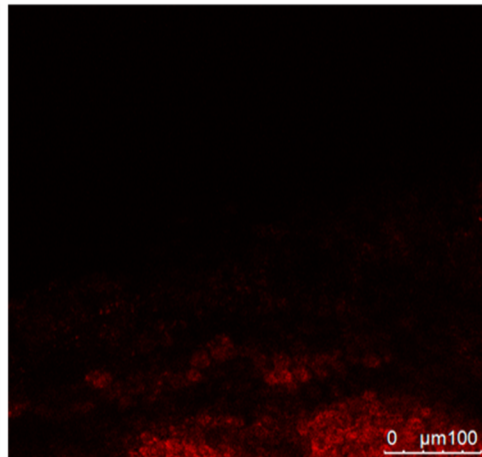

Bright

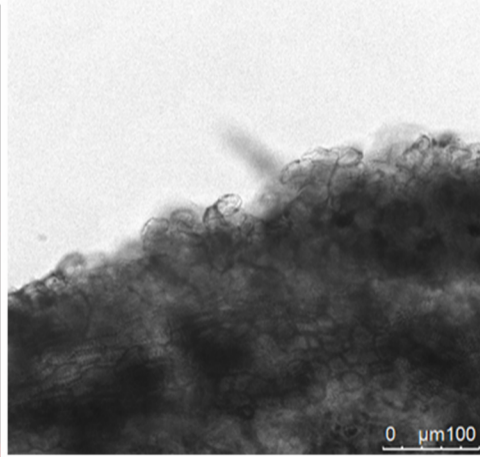

Merge

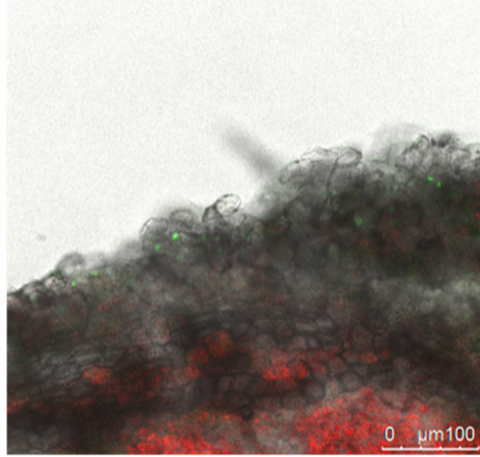

Supplement: Supplementary file 3 — Additional file 3: Figure S3. EdU staining of 84K poplar leaves after 6 days of differentiation culture. The picture shows the images under four channels of EdU, Chloroplast, Bright and Merge. The cells in S phase of cell cycle emit green fluorescence. [file 13007_2023_994_MOESM3_ESM.pdf]

A

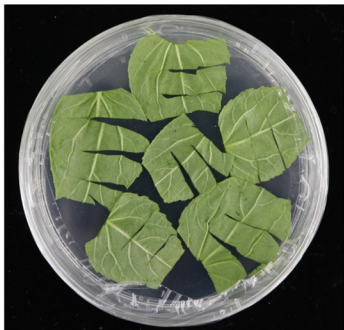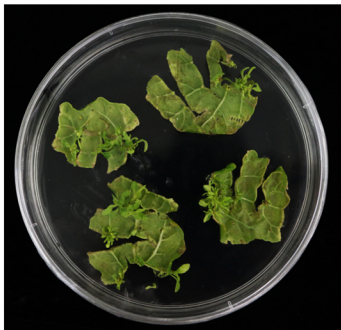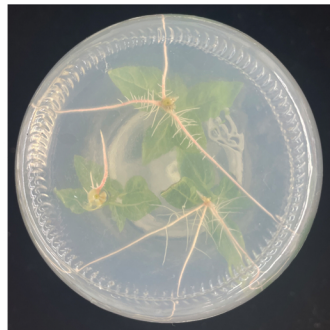

B

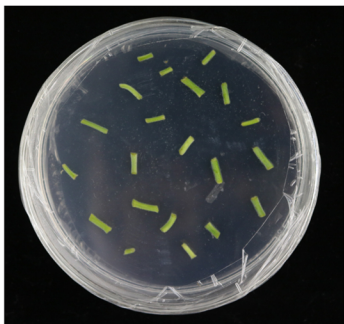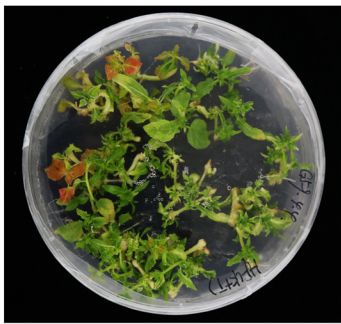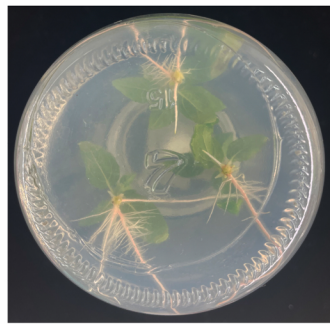

C

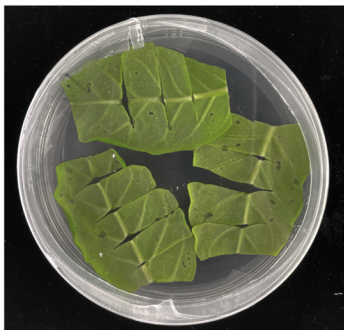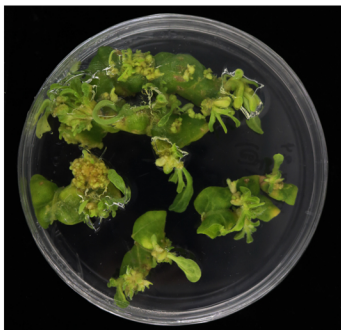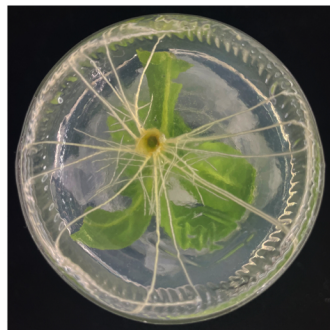

Supplement: Supplementary file 4 — Additional file 4: Figure S4. Regeneration of different receptor materials after treatment with Agrobacterium tumefaciens. (A) Phenotypic characteristics of 84K poplar leaves cultured on differentiation medium, budding and rooting. (B) Phenotypic characteristics of 84K poplar stem segments cultured on differentiation medium, budding and rooting. (C) Phenotypic characteristics of tobacco leaves cultured on differentiation medium, budding and rooting. [file 13007_2023_994_MOESM4_ESM.pdf]

A

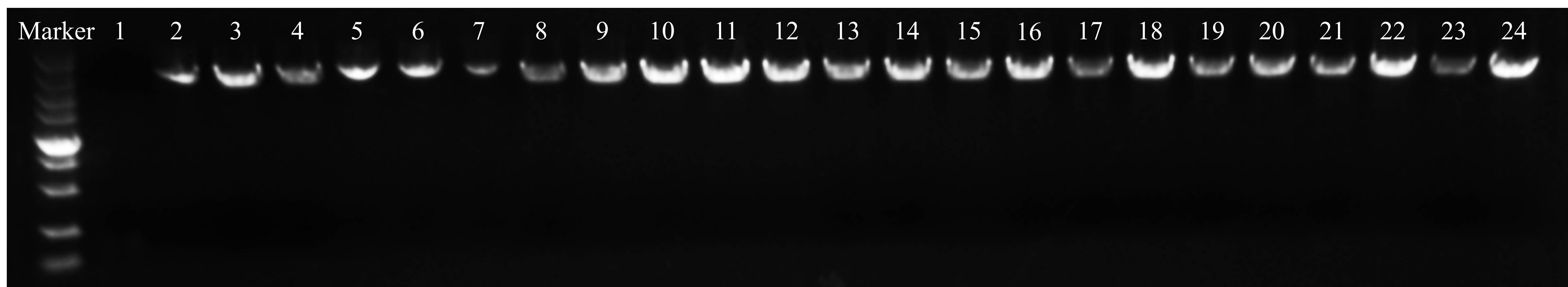

B

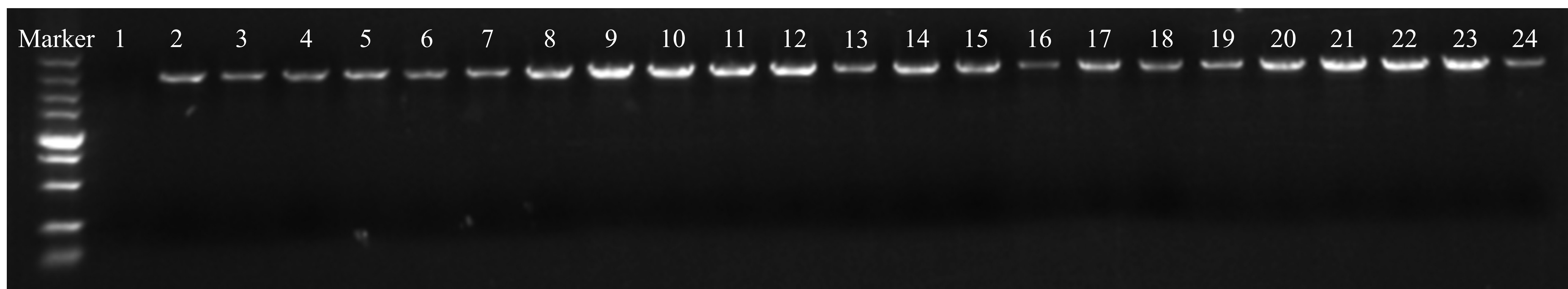

C

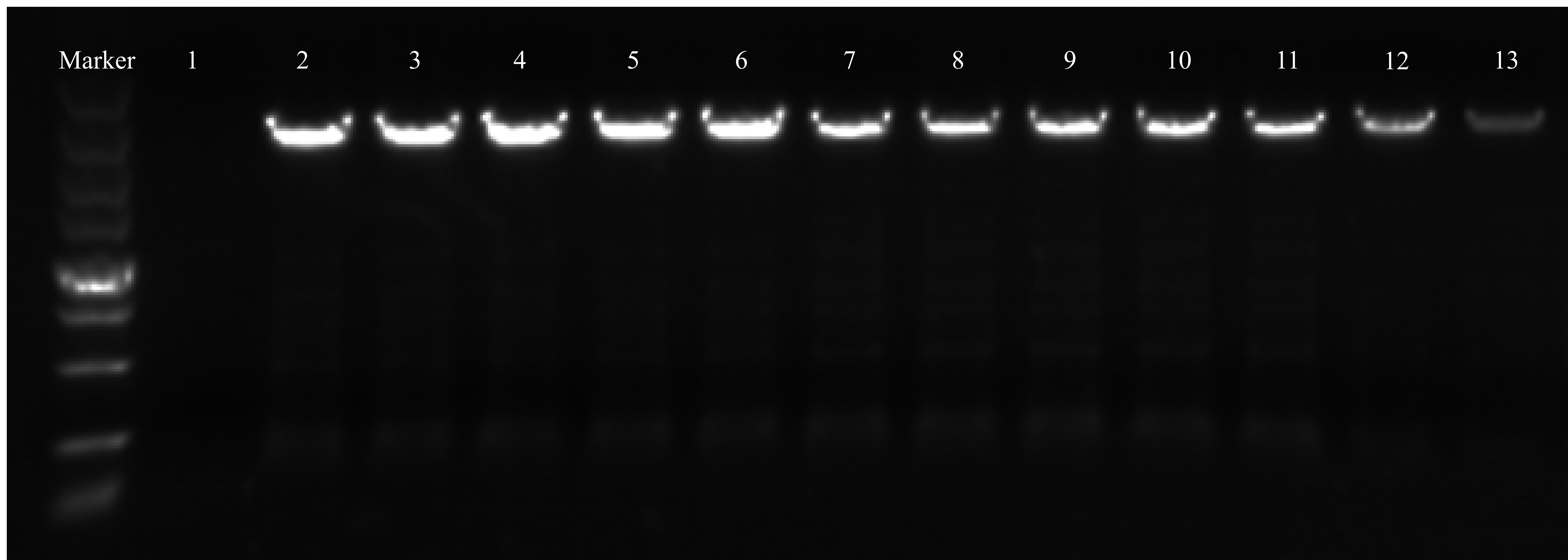

Supplement: Supplementary file 5 — Additional file 5: Figure S5. Detection of positive plantlets obtained from genetic transformation after differentiation culture of different receptor materials for different times. (A) PCR detection of genetic transformation positive seedlings of 84K poplar leaves, this testing experiment being repeated at least three times with similar results; 1: wild-type poplar; 2: plasmid; 3-24: transgenic plants. (B) PCR detection of genetic transformation positive seedlings of 84K poplar stem segments, this testing experiment being repeated at least three times with similar results; 1: wild-type poplar; 2: plasmid; 3-24: transgenic plants. (C) PCR detection of genetic transformation positive seedlings of tobacco leaves, this testing experiment being repeated at least three times with similar results; 1: wild-type tobacco; 2: plasmid; 3-13: transgenic plants. [file 13007_2023_994_MOESM5_ESM.pdf]
